# Supplementary material for: Distinct place cell dynamics in CA1 and CA3 encode experience in new environments
Source: Nat Commun. 2021 May 20;12:2977. doi: 10.1038/s41467-021-23260-3 (PMC8137926; doi:10.1038/s41467-021-23260-3)
Supplement: Supplementary file 1 — Supplementary Information [file 41467_2021_23260_MOESM1_ESM.pdf]

Distinct place cell dynamics in CA1 and CA3 encode experience in new environments

Supplementary figures

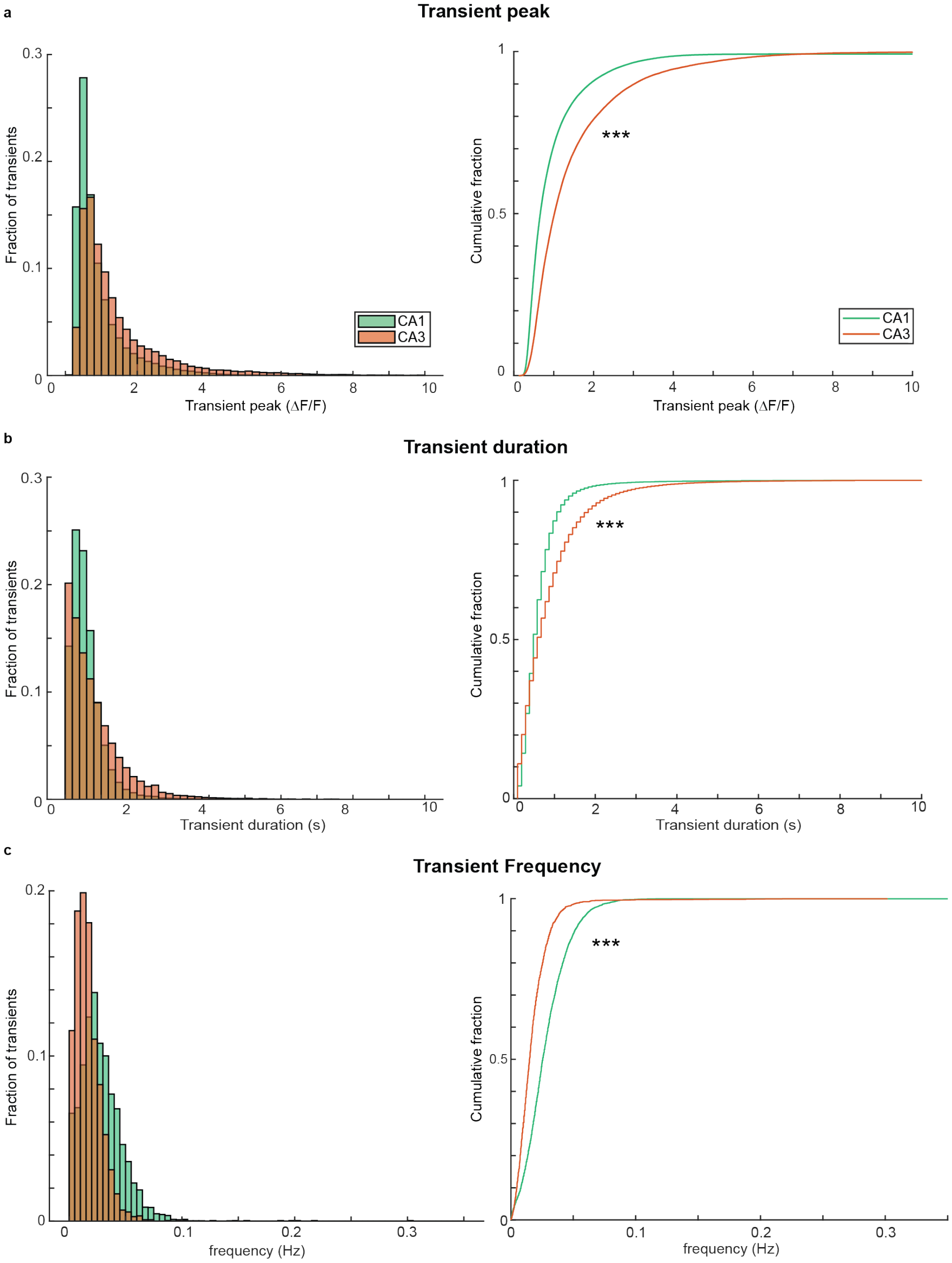

Supplementary Fig. 1 Calcium transient properties in CA1 and CA3. a, Left, histogram of the Calcium transient peaks for CA1 (green) and CA3 (orange). Right, the corresponding cumulative fraction plot. Wilcoxon rank sum test, two-sided, ***, $P < 1 \times 10^{-100}$. **b,** Left, histogram of the Calcium transient durations calculated at 50% of the peak. Right, the corresponding cumulative fraction plot. Wilcoxon rank sum test, two-sided, ***, $P < 1 \times 10^{-100}$. **c,** Left, histogram of Calcium transient frequencies. Right, the corresponding cumulative fraction plot. Wilcoxon rank sum test, two-sided, ***, $P < 1 \times 10^{-100}$.

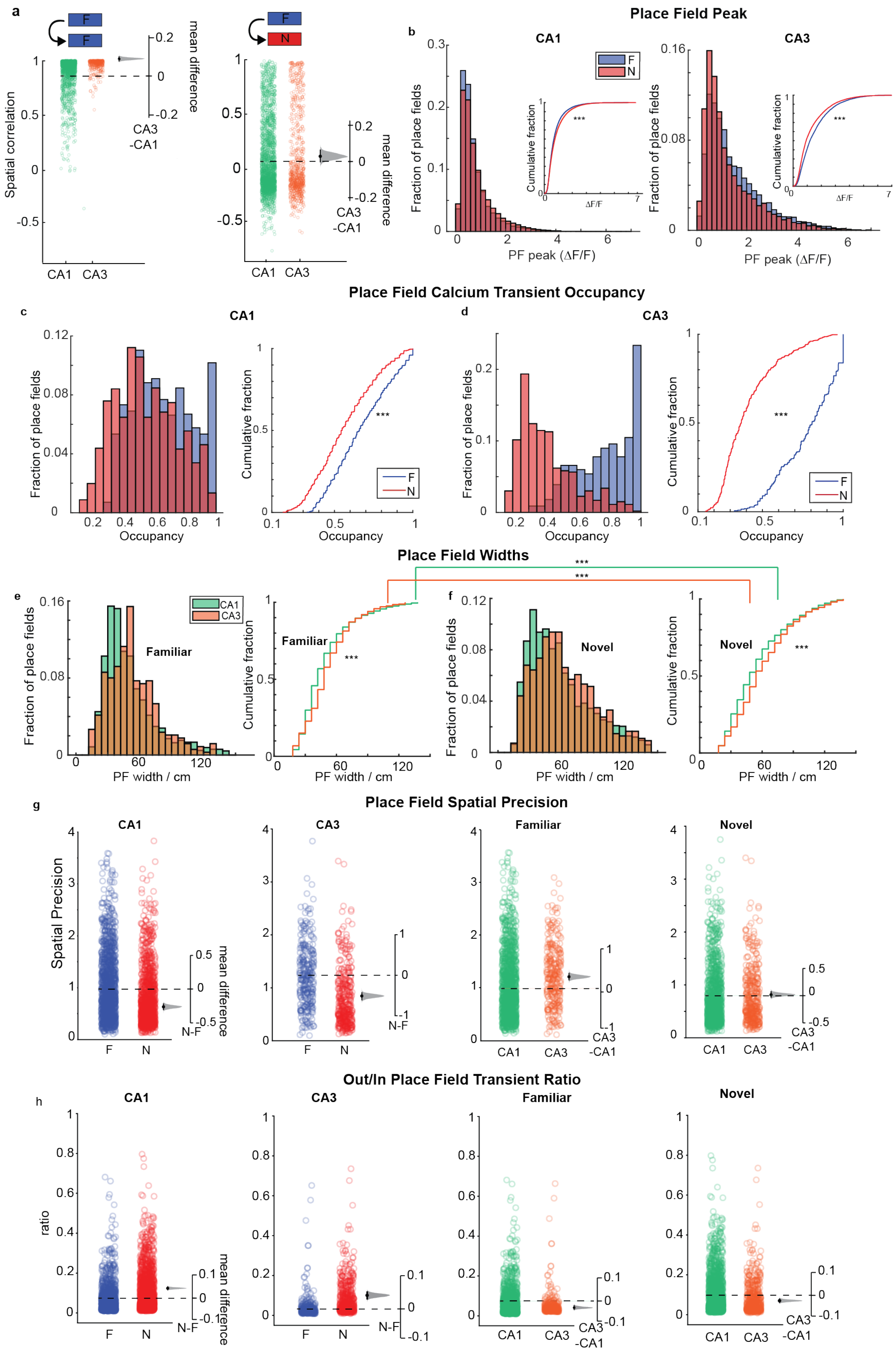
a
F
F
N
Spatial correlation
mean difference
CA3
-CA1
CA1
CA3
Place Field Peak
b
CA1
CA3
Fraction of place fields
Cumulative fraction
***
PF peak (ΔF/F)
ΔF/F
Place Field Calcium Transient Occupancy
c
d
Occupancy
Place Field Widths
e
f
Familiar
Novel
PF width / cm
Place Field Spatial Precision
g
Spatial Precision
N-F
Out/In Place Field Transient Ratio
h
ratio

Supplementary Fig. 2 Place field properties in familiar and novel environments in CA1 and CA3. **a,** Pearson's correlation coefficient of each cell's mean place field (PF) between CA1 and CA3 within familiar (F) sessions (left) and between F and novel (N) sessions (right). Bootstrapped mean difference (Δ) shown on the right of each plot. Note CA3 PFs are much more stable within F than CA1 PFs. **b,** Histogram of each PFs peak activity for CA1 (left) and CA3 (right) in F (red) and N (blue). Insets show cumulative fraction plots. Wilcoxon rank sum test, two-sided, ***, $P < 0.001$; CA1, $P = 4.5 \times 10^{-56}$, CA3, $P = 8.4 \times 10^{-79}$. **c,** Left, histogram of PF Calcium transient occupancy (percentage of laps with PF activity) in F (red) and N (blue) in CA1. Right, corresponding cumulative fraction plot. Wilcoxon rank sum test, two-sided, ***, $P < 0.001$, $P = 4.5 \times 10^{-56}$. **d,** Same plots as (c) in CA3, $P = 3.1 \times 10^{-95}$. Note the striking difference in transient occupancy in N versus F in CA3. The lower transient occupancy in N is largely due to delayed PF formation in N in CA3 (See Fig. 2). **e,** Histogram of each PFs width in CA1 (green) and CA3 (orange) in the familiar environment. Right, corresponding cumulative fraction plot. Wilcoxon rank sum test, two-sided, ***, $P < 0.001$, CA1-CA3 F, $P = 7.1 \times 10^{-4}$, CA1-CA3 N, $P = 4.9 \times 10^{-5}$, CA1 F-N, $P = 3.2 \times 10^{-15}$, CA3F-N, $P = 2.6 \times 10^{-6}$. **f,** Same plots as (e) but in N. **g,** Spatial precision of each PF in CA1 (left) and CA3 (middle left) in F (blue) and N (red). Same data are also compared across CA1 and CA3. Bootstrapped mean difference (Δ) shown on the right of each plot. Note that PFs in N are less precise than in F in both CA1 and CA3, and CA3 PFs in F are more precise than CA1 PFs in F. **h,** Ratio of out versus in PF activity for CA1 (left) and CA3 (middle left) in F (blue) and N (red). Same data are also compared in F (middle right) and N (right) across CA1 and CA3. Bootstrapped mean difference (Δ) shown on the right of each plot. Note that PFs in N have more out-of-field firing than F in both regions but CA3 has less out-of-field firing than CA1.

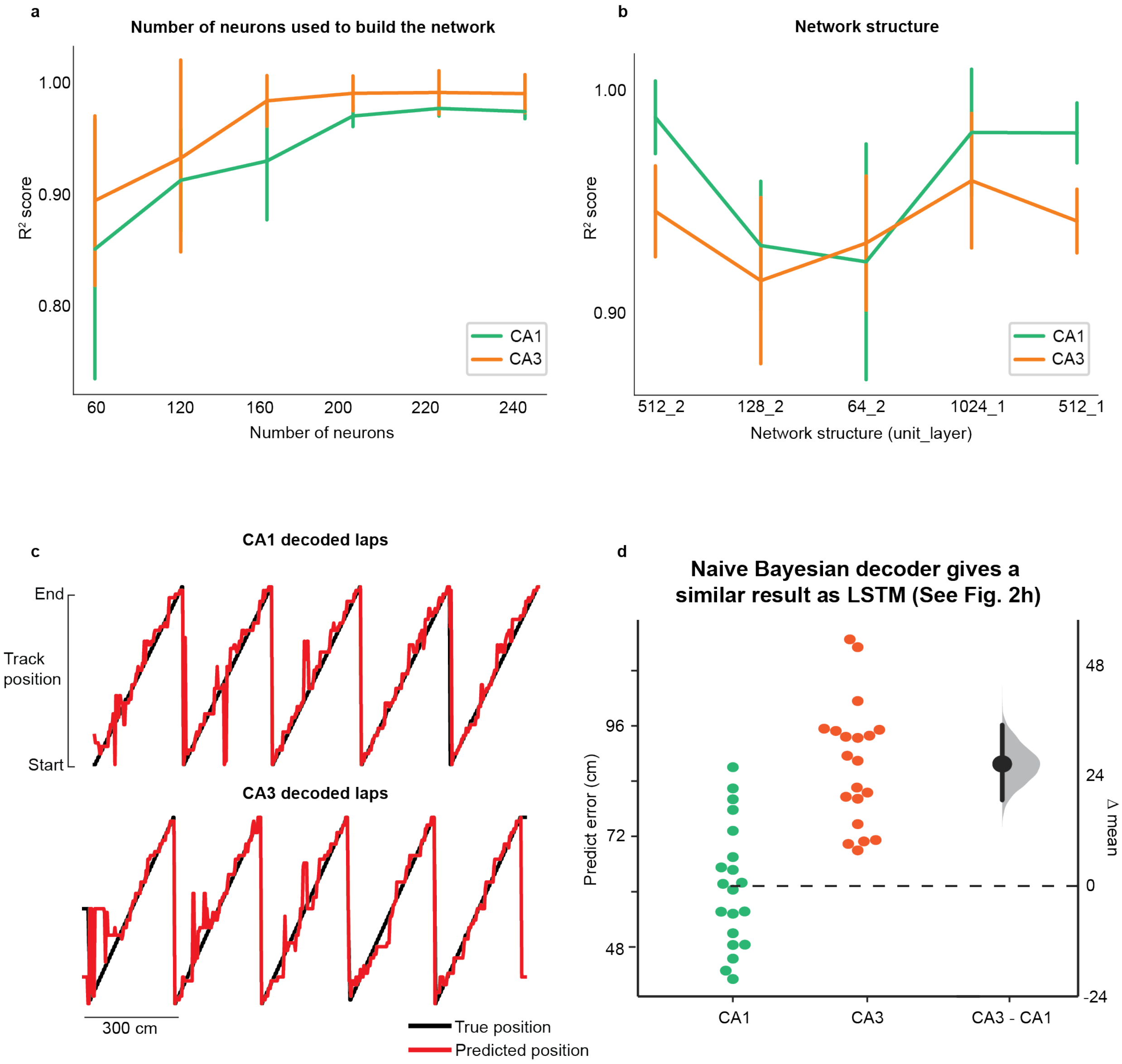

Supplementary Fig. 3 Long-Short-Term-Memory (LSTM) and Bayesian decoding of animal position. a, Mean LSTM decoder performance ± SEM versus the number of place cells that were used to build the decoder. The network structure for all the decoders are the same, a one-layer 1024-unit neuronal network. **b,** Mean decoding performance ± SEM versus the network structure for the decoder. All decoders were trained with 200 place cells. **c,** Example mouse showing true track position (black) on laps 36-40 and the predicted position (red) decoded by a Naïve Bayesian decoder. Note the LSTM decoder (Fig. 2f) did a much better job than the Bayesian decoder. **d,** Average predicted error for the first lap in the novel environment for CA1 and CA3. Each dot represents the decoding error for one decoder trial built based on the activity of 200 randomly chosen place cells from CA1 (green) or CA3 (orange) data. *n* = 20 decoder trails. Bootstrapped mean difference (Δ) shown on the right of each plot.

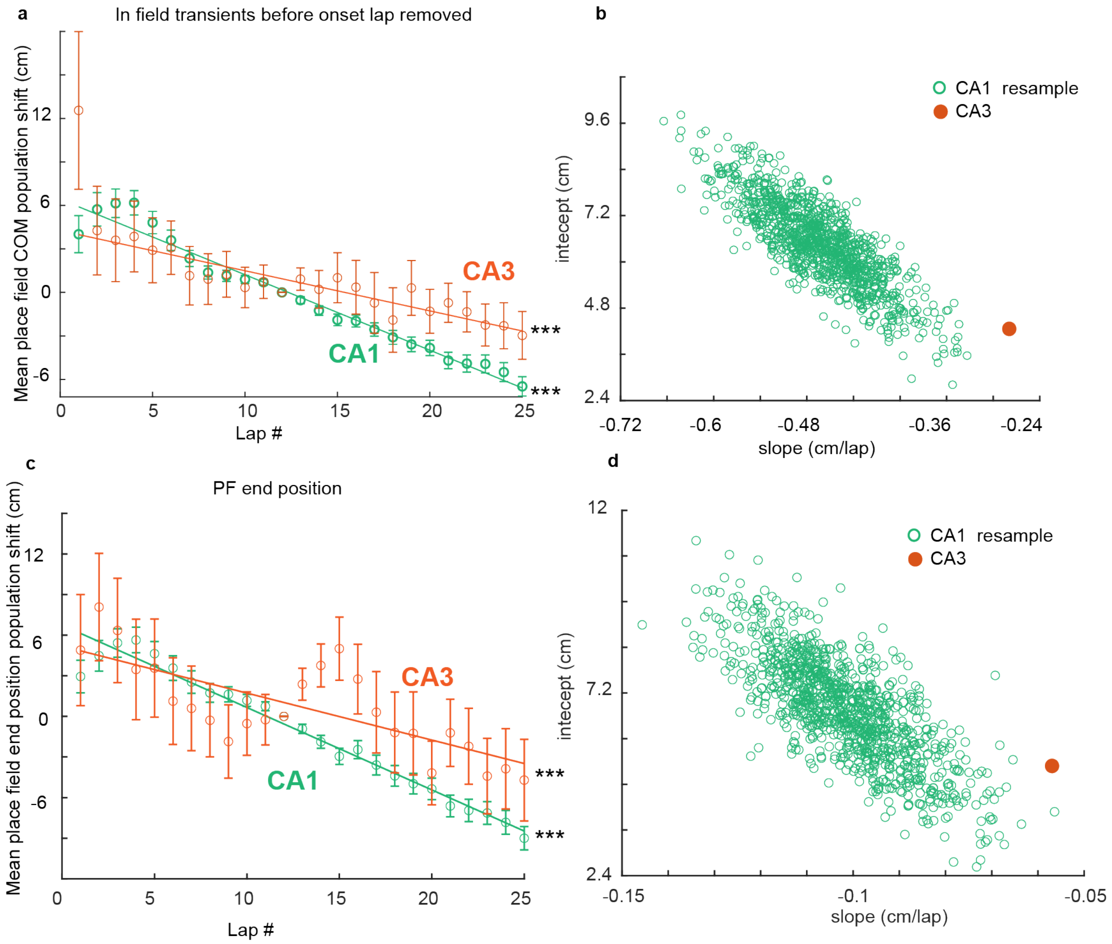

Supplementary Fig. 4 Population backward shifting is still observed when transients that occur prior to place field emergence are removed and when place field position on each lap is defined by the location of the calcium transient end point. a, Population shift of COM. Mean ± SEM from all place fields (PFs) using a 5-lap sliding average. COM difference is relative to lap 12. All in-field transients that occurred prior to PF onset lap have been removed. Linear regression, F-test, ***, $P < 0.001$, CA1, $P < 1 \times 10^{-100}$, CA3, $P = 1.5 \times 10^{-7}$. **b**, Resampling analysis (1000 resamples) associated to (a) shows CA1 backward shifting is still significantly faster than in CA3. **c**, Population PF shift on each lap not defined by the COM but by the location of the end of the calcium transient. Mean ± SEM from all PFs using a 5-lap sliding average. COM difference relative to lap 12. This shows that the location of the end position of the PF shifts backward in both CA1 and CA3. Linear regression, F-test, ***, $P < 0.001$, CA1, $P < 1 \times 10^{-100}$, CA3, $P = 9.9 \times 10^{-6}$. **d**, same as (b) but associated to (c): CA1 population shifting slopes are still significantly more negative than CA3 ($P = 0.001$).

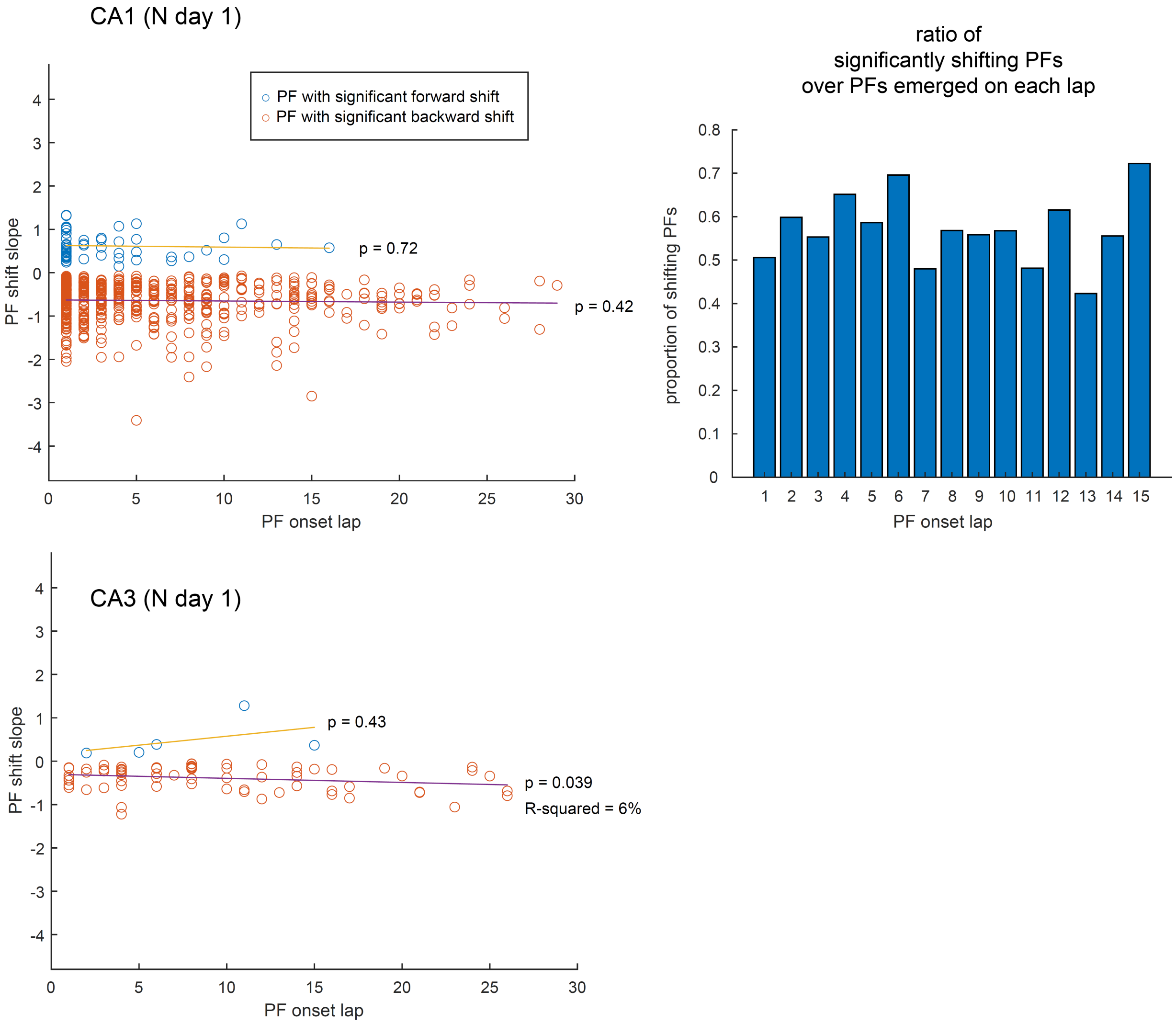

Supplementary Fig. 5 Lap-by-lap shifting is not related to the timing of PF emergence Place field (PF) shift slope is defined as in Fig. 3c. Only significantly shifting PFs (regression $P < 0.05$) are included. No correlation between PF onset lap and the amplitude of their shifting dynamics is observed in CA1, and only weakly in CA3 where the sample size is low. Note that, in CA1, the higher number of shifting PFs with early onset is simply due to the higher number of PFs emerging in early laps. The proportion of shifting PFs (Right panel) stays stable throughout the first 15 laps (later laps not shown because number of shifting PFs < 10). Linear regression, F-test.

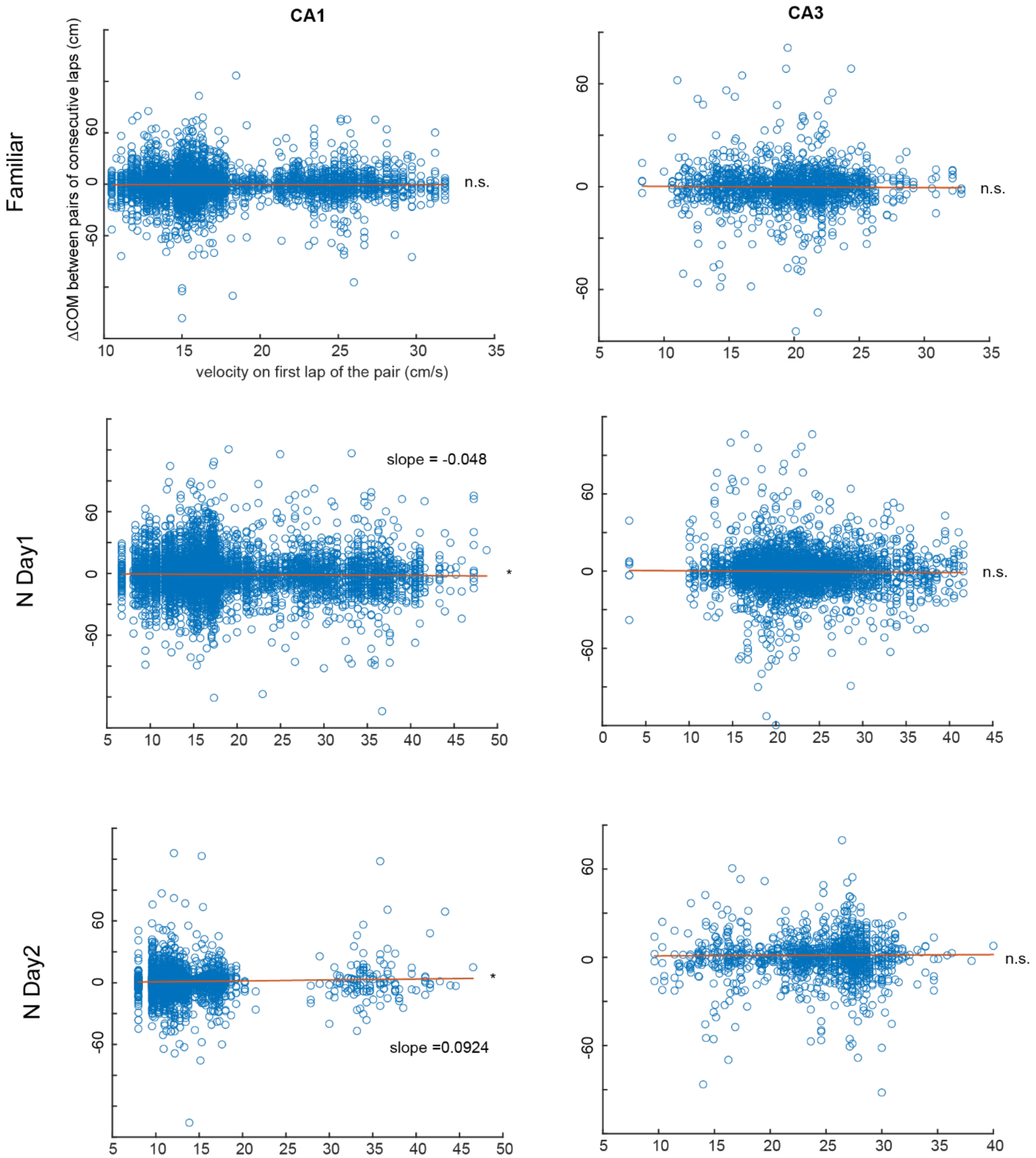

Supplementary Fig. 6 Relationship between place field shifting and lap velocity. For all place fields (PFs), all differences in COM position between consecutive laps (ΔCOM) are plotted against the velocity on the first lap of the pair (similar results when velocity on second lap is used instead). This analysis does not reveal an obvious relationship between velocity and shifting and clearly shows that large lap-to-lap shifts are not due to higher velocities. Linear regression, F-test.

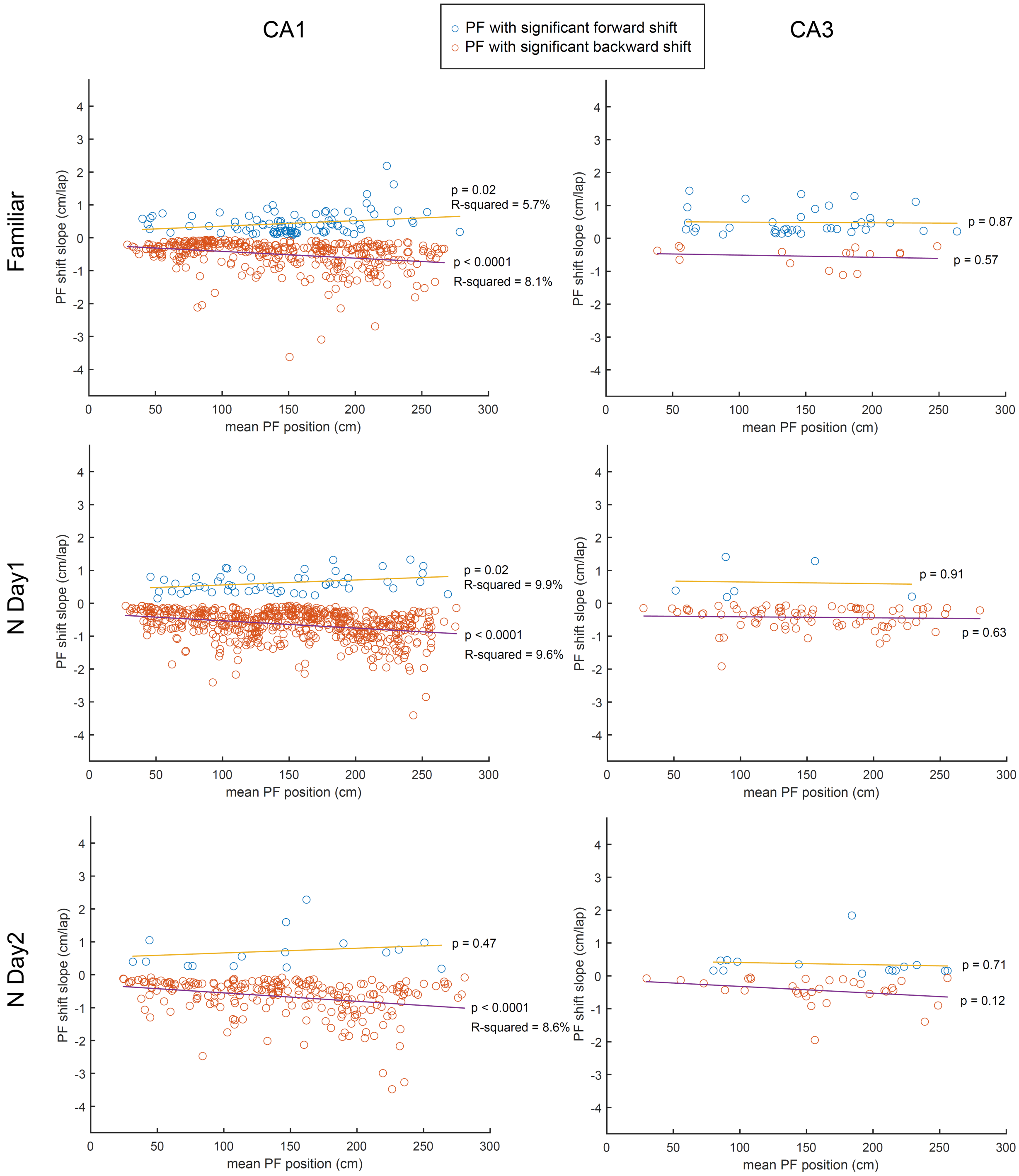

Supplementary Fig. 7 Place field shifting is weakly correlated to place field position. Each data point corresponds to the shifting slope of a single place field (PF) from the regression analysis on the PF COM lap-by-lap position (see Fig. 3c). Only PFs with regression *P*-value < 0.05 are included. In CA1, PF shifting amplitude is weakly but significantly correlated with the PF mean COM position in each recording day and environment. PFs with a large forward or backward shift are biased toward the end of the track. No correlation is detected in CA3 but the number of shifting PFs is much lower. Linear regression, F-test.

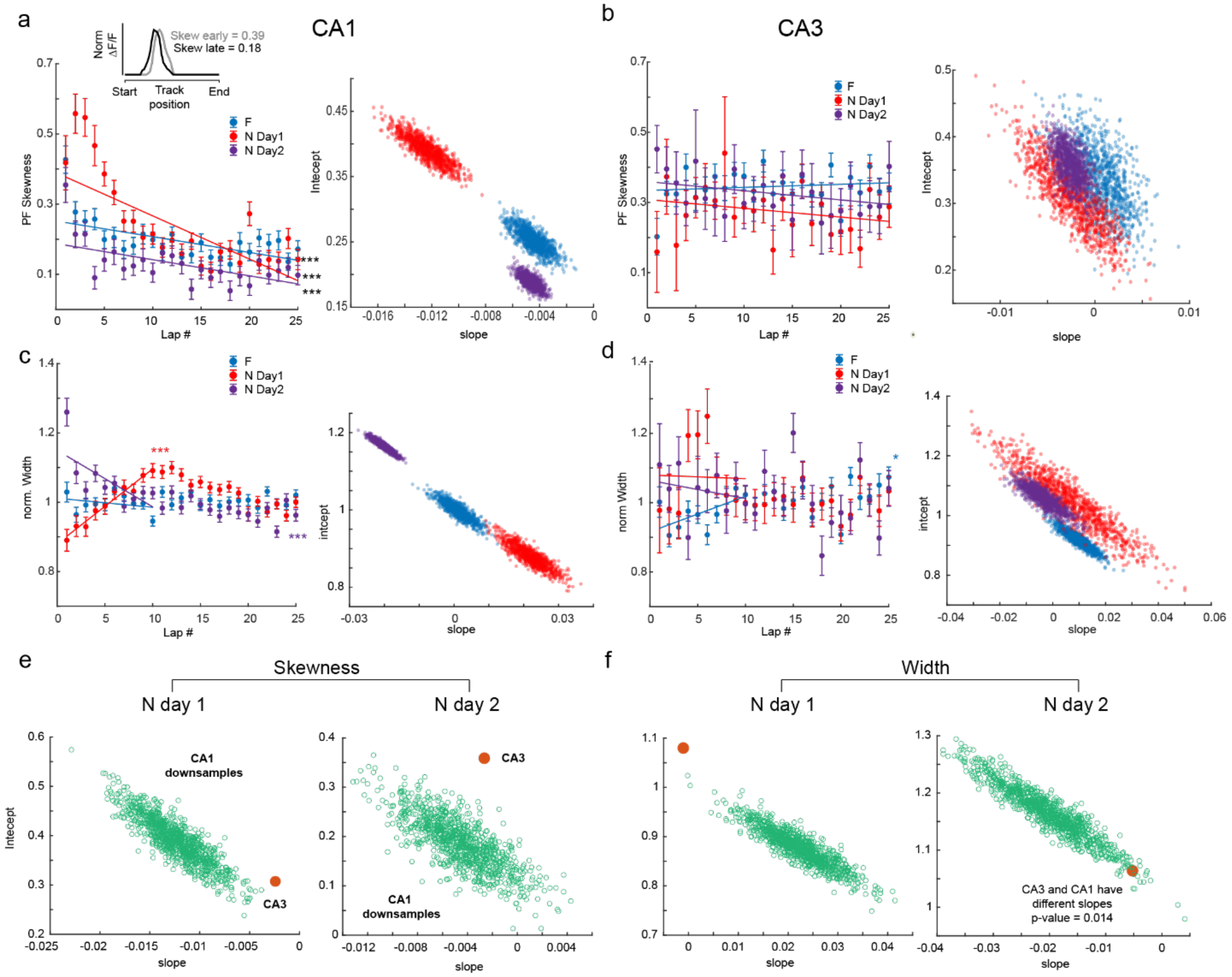

Supplementary Fig. 8 Lap-by-lap change of place field skewness and width. a, Left, Lap-wise mean ± SEM skewness over all place fields (PFs) recorded in a given condition. Linear regression (on data points, not means) shows a decrease in positive skewness with experience, especially in N day 1. Inset: representative example PF that shifted backward, became less positively skewed and wider (early = lap 1-5 average fluorescence activity, late = lap 25-30 average). Right, resampling analysis as in Fig. 6c: slopes are significantly different from N day 1 to N day 2 and stay similar in a very familiar environment (F). Overall, these results suggest that PFs become less skewed over the first 10 laps in N and seem to stabilize afterwards, with slow population change upon re-exposure. Linear regression, F-test, ***, $P < 0.001$, F, $P = 9.09 \times 10^{-10}$, N Day1, $P = 9.59 \times 10^{-39}$, N Day2, $P = 9.06 \times 10^{-8}$. **b,** same as (a) for CA3. Distribution spread is wide, suggesting less homogeneity in the population of PFs. Overlap in distributions show dynamics do not change significantly with familiarization. Linear regression, F-test, F, $P = 0.40$, N Day1, $P = 0.28$, N Day2, $P = 0.23$. **c-d,** Same as (a) for the lap-wise PF width normalized to width averaged over all laps for each PF (i.e. 1 means that the width is the same as the average PF width). This normalization controls for large variations in individual PF width and allows direct comparison with Mehta et al. 1997[1]. In CA1, PF width increases over first 10 laps in N but decreases on second day and is stable in F. CA3 does not show clear population dynamics, although PFs become more homogeneous with familiarization across days. Linear regression fit to data points from the

first 10 laps only. Linear regression, F-test, ***, $P < 0.001$. CA1 : F, $P = 0.20$, N Day1, $P = 3.88 \times 10^{-16}$, N Day2, $P = 1.12 \times 10^{-10}$; CA3: F, $P = 0.001$, N Day1, $P = 0.90$, N Day2, $P = 0.50$. **e-f,** Resampling analysis as described in Fig. 3e. CA3 and CA1 PF width and skewness dynamics (slopes) are significantly different on day 1, but only widths are different on day 2.

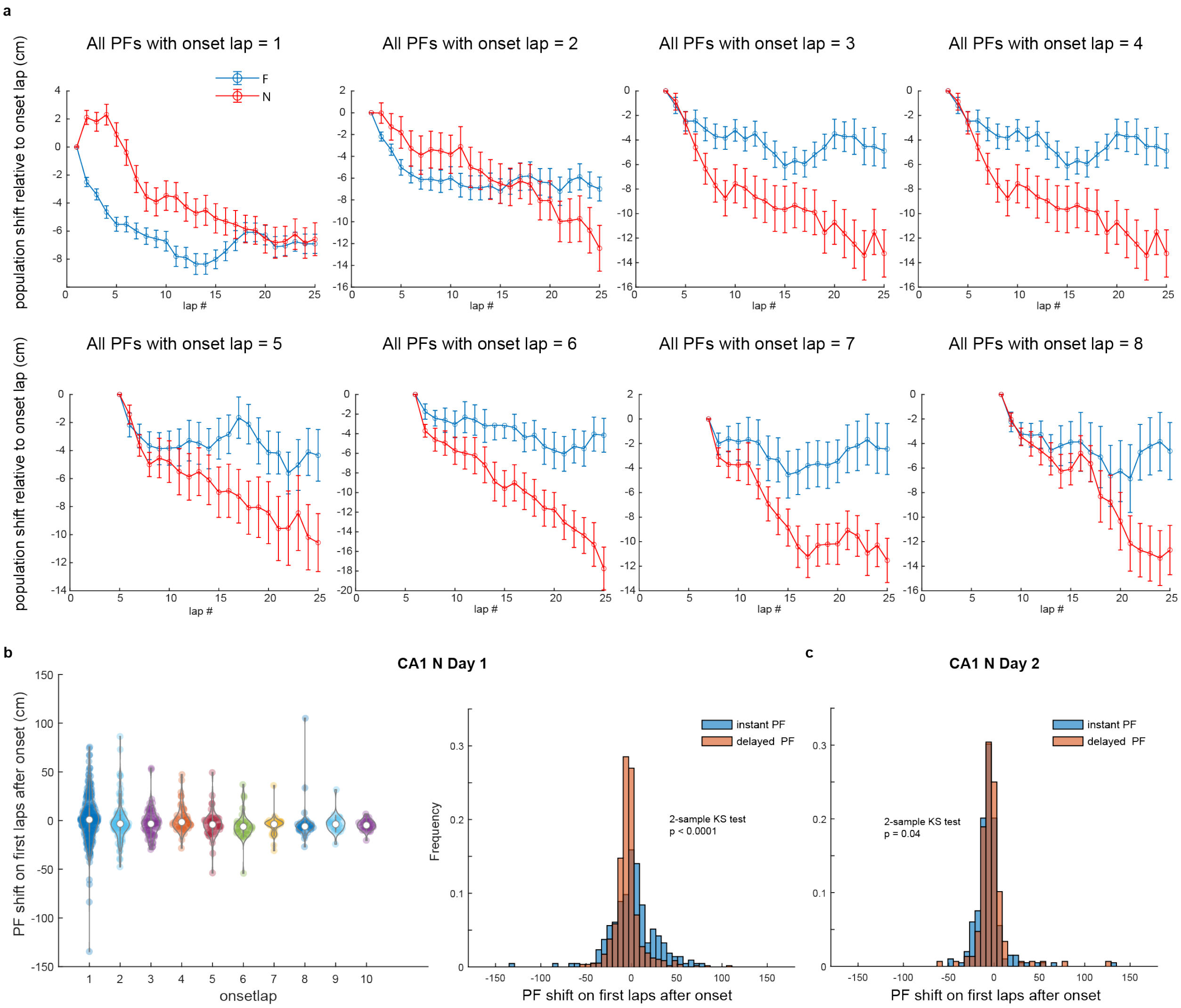

Supplementary Fig. 9 CA1 forward shifting during first laps is driven by instant-onset place fields. a, Mean ± SEM, for all place fields (PFs) of 5-lap rolling average center of mass (COM) difference relative to onset lap, in novel (N) and familiar (F) environments. Forward shifting of the population after onset is only seen for instant PFs (onset lap = 1). **b,** Left, difference between 5-lap rolling average COM at onset lap and next lap, for each PFs, as a function of onset lap (not showing onset lap > 10). The median is above 0 for instant PFs, not the other groups. Right, same as b but combining all delayed PFs together for comparison with instant PFs. Distributions are significantly different, with more early forward shifting PFs in the instant PFs group. **c,** The effect seen in b disappears on day 2. Distributions are only mildly different and not because of a higher number of early forward shifting PFs in the instant onset group.

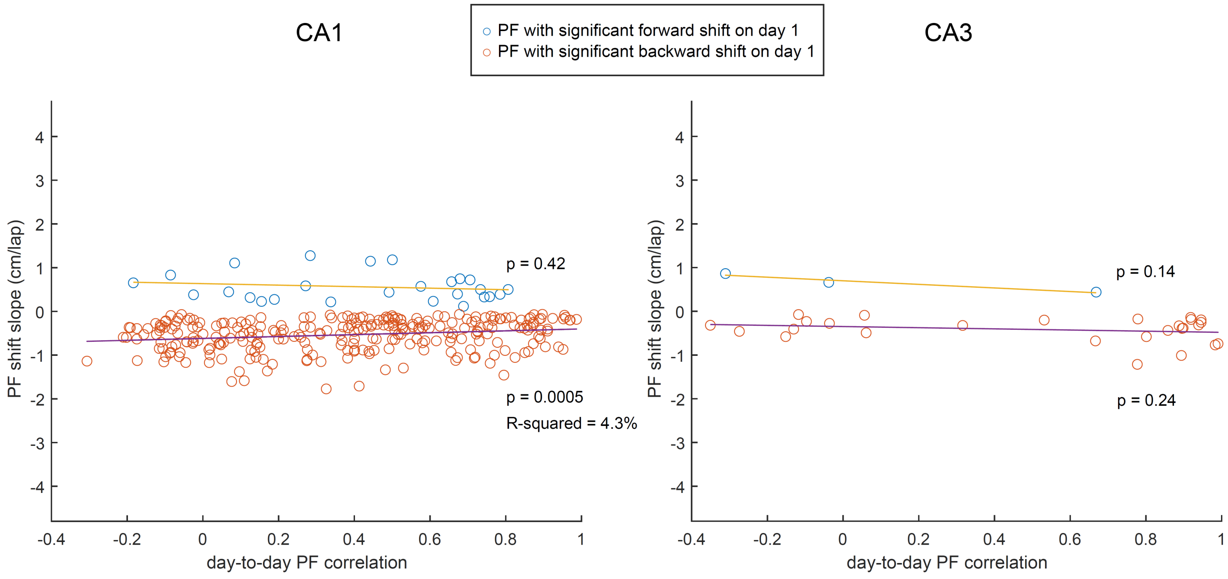

Supplementary Fig. 10 CA1 place field stability across days is correlated to lap-by-lap stability on day 1. Place field (PF) shift slope is defined as in Fig. 3c. Only significantly shifting PFs (regression $P < 0.05$) are included. In CA1, there is a weak correlation (linear regression) between day-to-day stability and day 1 lap-by-lap dynamics for backward shifting PFs, suggesting that PFs that are stable across days are also more stable from lap-to-lap on day 1. Although not significant, this trend is seen for forward shifting PFs too. In CA3, PFs do not shift as much (see Fig. 3), resulting in a low sample size and no apparent correlation. Linear regression, F-test.

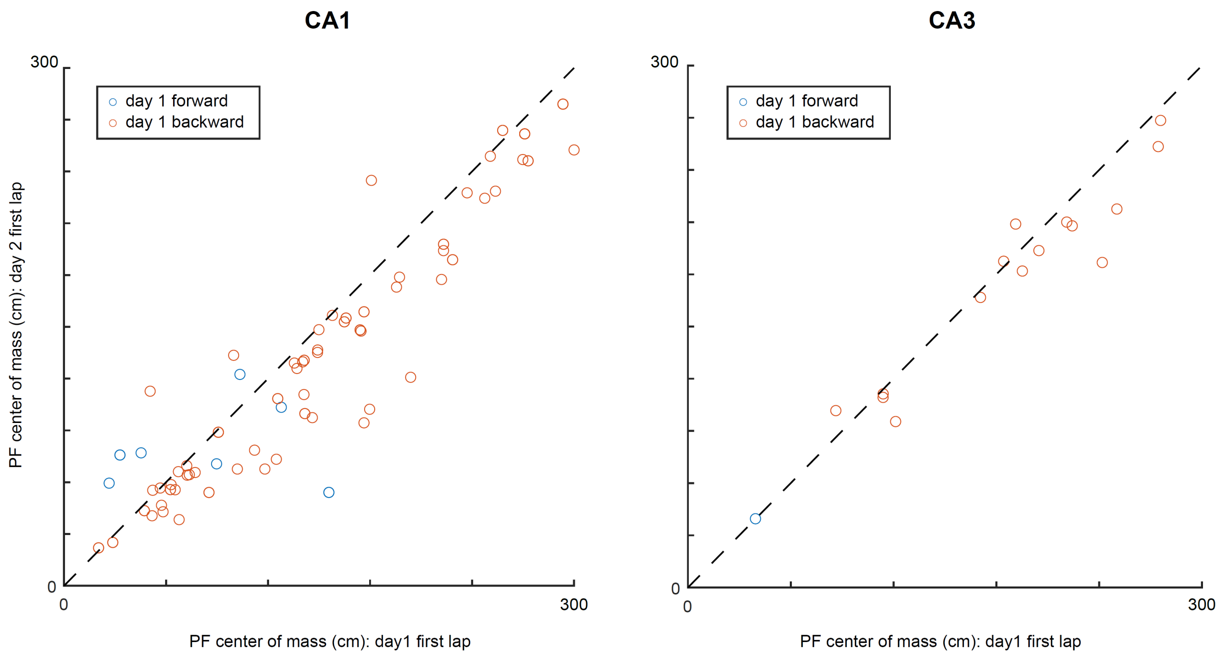

Supplementary Fig. 11 On day 2, shifting place fields do not necessarily reset to their exact initial position on day 1. Place field (PF) shift slope is defined as in Fig. 3c. Selected PFs (day-to-day correlation > 0.5 and significant shifting on day 1) are the same as in Fig. 5b.

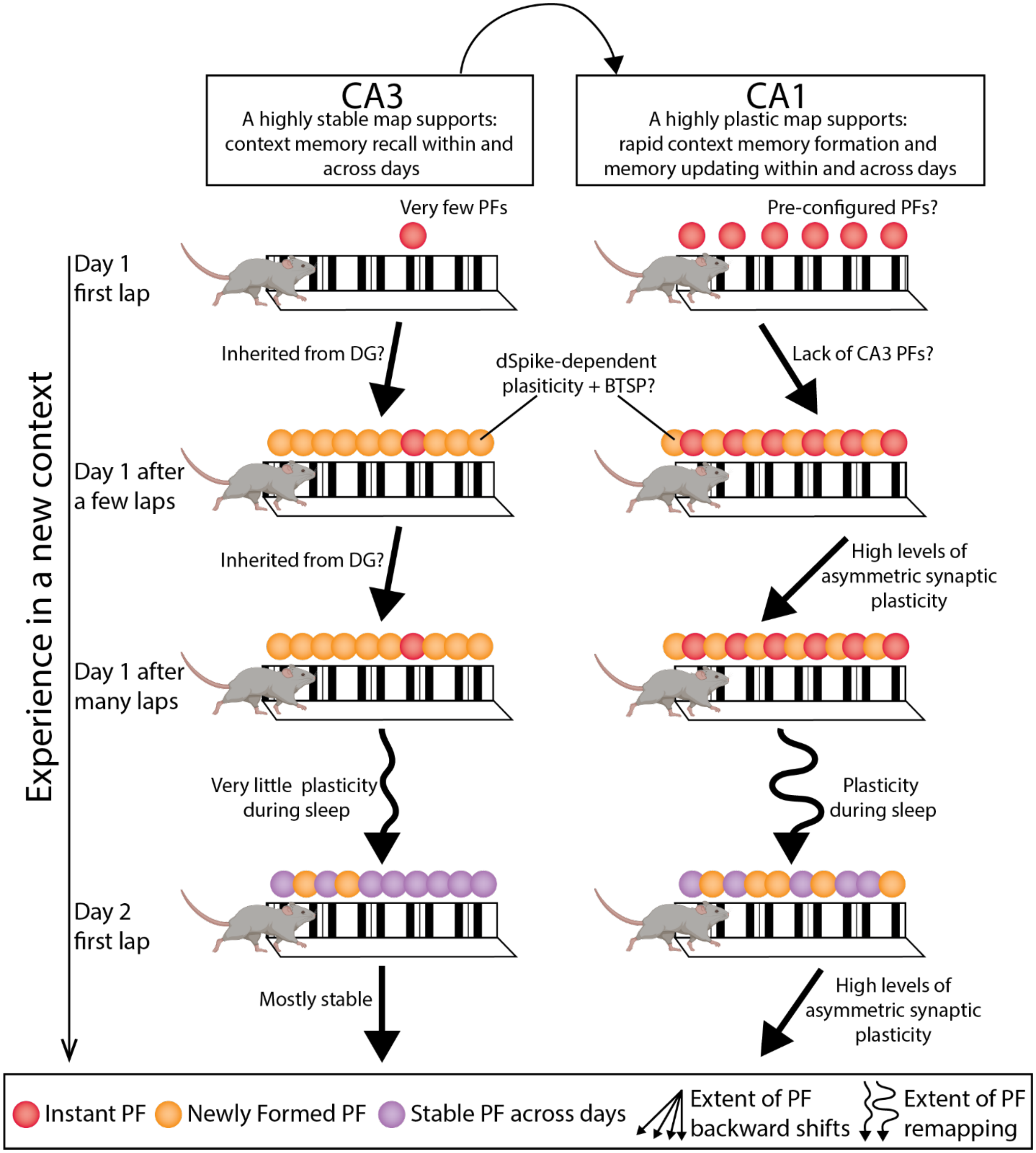

Supplementary Fig. 12 Conceptual model. In a new context, place fields (PFs) in CA1 instantly appear on the first traversal (red circles). These PFs do not require experience-dependent synaptic plasticity and so are likely pre-configured[2]. Instant PFs are lacking in CA3. Instant PFs in CA1 initially shift forward for the first 1-4 laps, possibly due to the lack of CA3 PFs. Within the first few laps in the new context, new PFs appear in both CA1 and CA3 (orange circles). We partially know how these PFs form in CA1, but it remains unknown whether the same mechanisms are at play in CA3. In CA1, these PFs form through local dendritic spikes (dSpikes) that induce synaptic

potentiation through an NMDA receptor mechanism[3]. This process occurs during the silent period when these cells are not firing. On the lap where the PF first appears, in some or many of these cells, behavioral timescale synaptic plasticity (BTSP) further strengthens synapses more globally throughout the neuron through burst firing associated with plateau potentials generated in the dendrites by coincident input from CA3 and Entorhinal cortex[4]. Throughout experience the newly formed PFs shift backwards and develop NMDA-dependent negative skewness in CA1 likely through asymmetric synaptic plasticity at the CA3-CA1 synapse. CA3 PFs show only a small amount of backward shifting and no increase in negative skewness, suggesting they may inherit their shifts from their inputs. Across days, PFs in CA1 undergo partial remapping, which likely involves synaptic plasticity that occurs during sleep when place cell sequences are known to be reactivated. CA3 PFs are much more stable across days. Throughout experience on day 2, a reduced but still very significant level of backward shifting occurs in CA1 with even less backward shifting in CA3. These PF dynamics in CA1 and CA3 likely support distinct roles in memory processing. We suggest that the CA1 rapidly forms a memory of a new context that supports single trial learning, and continuously updates this memory throughout ongoing experience. This updating - in the form of backward shifting PFs - may enable the CA1 to predict the near future regarding where in the context the animal is about to visit. The partial remapping across days in CA1 may also be another form memory updating by separating memory representations of experiences that occur in the same context on different days or at different times. On the other hand, animals need to recognize where they are and recall whether they are in a context they have experienced before. CA3 PFs seem to support this memory process by maintaining stable PFs within and across days. Picture of rodent created with BioRender.com.

References

1. Mehta, M. R., Barnes, C. A. & McNaughton, B. L. Experience-dependent, asymmetric expansion of hippocampal place fields. *Proc. Natl. Acad. Sci. USA* **94**, 8918–8921 (1997).
2. Dragoi, G. & Tonegawa, S. Preplay of future place cell sequences by hippocampal cellular assemblies. *Nature* **469**, 397–401 (2011).
3. Sheffield, M. E. J., Adoff, M. D. & Dombeck, D. A. Increased prevalence of calcium transients across the dendritic arbor during place field formation. *Neuron* **96**, 490–504 (2017).
4. Bittner, K. C., Milstein, A. D., Grienberger, C., Romani, S. & Magee, J. C. Behavioral time scale synaptic plasticity underlies CA1 place fields. *Science* **357**, 1033–1036 (2017).
