# Supplementary material for: Distinct place cell dynamics in CA1 and CA3 encode experience in new environments
Source: Nat Commun. 2021 May 20;12:2977. doi: 10.1038/s41467-021-23260-3 (PMC8137926; doi:10.1038/s41467-021-23260-3)
Supplement: Supplementary file 3 — Reporting Summary [file 41467_2021_23260_MOESM3_ESM.pdf]

nature research

Corresponding author(s): Mark Sheffield

Last updated by author(s): 2021/03/28

Reporting Summary

Nature Research wishes to improve the reproducibility of the work that we publish. This form provides structure for consistency and transparency in reporting. For further information on Nature Research policies, see our Editorial Policies and the Editorial Policy Checklist.

Statistics

For all statistical analyses, confirm that the following items are present in the figure legend, table legend, main text, or Methods section.

n/a	Confirmed	
☐	☒	The exact sample size (n) for each experimental group/condition, given as a discrete number and unit of measurement
☐	☒	A statement on whether measurements were taken from distinct samples or whether the same sample was measured repeatedly
☐	☒	The statistical test(s) used AND whether they are one- or two-sided<br>*Only common tests should be described solely by name; describe more complex techniques in the Methods section.*
☒	☐	A description of all covariates tested
☐	☒	A description of any assumptions or corrections, such as tests of normality and adjustment for multiple comparisons
☐	☒	A full description of the statistical parameters including central tendency (e.g. means) or other basic estimates (e.g. regression coefficient) AND variation (e.g. standard deviation) or associated estimates of uncertainty (e.g. confidence intervals)
☐	☒	For null hypothesis testing, the test statistic (e.g. F, t, r) with confidence intervals, effect sizes, degrees of freedom and P value noted<br>*Give P values as exact values whenever suitable.*
☐	☒	For Bayesian analysis, information on the choice of priors and Markov chain Monte Carlo settings
☐	☒	For hierarchical and complex designs, identification of the appropriate level for tests and full reporting of outcomes
☐	☒	Estimates of effect sizes (e.g. Cohen's d, Pearson's r), indicating how they were calculated

Our web collection on statistics for biologists contains articles on many of the points above.

Software and code

Policy information about availability of computer code

Data collection	VR environments were created using VIRMEn, an open source software for VR experiment design.<br>Imaging data collection was done using a laser scanning two-photon microscope (Neurolabware).<br>Mice behavior including treadmill running speed, position, and licking and imaging signal were collected using the PicoScope Oscilloscope (PICO4824, Pico Technology).
Data analysis	Calcium imaging data motion correction and ROI extraction was completed by customized MATLAB scripts (detail see methods).<br>All subsequent analyses were conducted with custom Matlab (2018a) and Python (3.7) codes, available at the following Github public repository: https://github.com/Candong/Distinct_CA1_CA3.

For manuscripts utilizing custom algorithms or software that are central to the research but not yet described in published literature, software must be made available to editors and reviewers. We strongly encourage code deposition in a community repository (e.g. GitHub). See the Nature Research guidelines for submitting code & software for further information.

Data

Policy information about availability of data

All manuscripts must include a data availability statement. This statement should provide the following information, where applicable:

- Accession codes, unique identifiers, or web links for publicly available datasets
- A list of figures that have associated raw data
- A description of any restrictions on data availability

The datasets generated during and/or analyzed during the current study are available on the Github public repository (https://github.com/Candong/Distinct_CA1_CA3).
The raw imaging data are available from the corresponding author upon reasonable request. Source data for each figure are provided as a spreadsheet.

Field-specific reporting

Please select the one below that is the best fit for your research. If you are not sure, read the appropriate sections before making your selection.

- [x] Life sciences
- [ ] Behavioural & social sciences
- [ ] Ecological, evolutionary & environmental sciences

For a reference copy of the document with all sections, see nature.com/documents/nr-reporting-summary-flat.pdf

Life sciences study design

All studies must disclose on these points even when the disclosure is negative.

Sample size	Sample size was based on these previous publications (Dombeck et al, 2007; Sheffield et al, 2017).
Data exclusions	Analysis related to PF COM, skewness and width excluded PFs that were clipped at the start and end of the track. These measures are artificially influenced by clipped PFs so they had to be excluded from this analysis.
Replication	Animals in this study came from multiple litters and were trained and recorded on two different microscope/VR set-ups during different times. The findings are consistent across individual animals and all replication attempts were successful.
Randomization	Mice were randomly chosen and the data was collected from multiple groups from different litters.
Blinding	All data collection and analysis was performed with automated software, with the same settings used across all animal subjects. Only one Investigator handled the animals, following the same procedure. This investigator was not blinded to CA1 or CA3 groups.

Reporting for specific materials, systems and methods

We require information from authors about some types of materials, experimental systems and methods used in many studies. Here, indicate whether each material, system or method listed is relevant to your study. If you are not sure if a list item applies to your research, read the appropriate section before selecting a response.

Materials & experimental systems

n/a	Involved in the study	
[x]	[ ]	Antibodies
[x]	[ ]	Eukaryotic cell lines
[x]	[ ]	Palaeontology and archaeology
[ ]	[x]	Animals and other organisms
[x]	[ ]	Human research participants
[x]	[ ]	Clinical data
[x]	[ ]	Dual use research of concern

Methods

n/a	Involved in the study	
[x]	[ ]	ChIP-seq
[x]	[ ]	Flow cytometry
[x]	[ ]	MRI-based neuroimaging

Animals and other organisms

Policy information about studies involving animals; ARRIVE guidelines recommended for reporting animal research

Laboratory animals	10-12 week old C57BL/6J wildtype (WT) male mice (4 for CA1 population imaging, Jackson Lab 000664) and 10-12 week old C57BL/6-Tg(Grik4-cre)G32-4Stl/J male mice (7 for CA3 population imaging, Jackson Lab, 006474) were used.
Wild animals	No wild animals were used in the study.
Field-collected samples	No field collected samples were used in the study.
Ethics oversight	University of Chicago Animal Care and Use Committee

Note that full information on the approval of the study protocol must also be provided in the manuscript.
